# Aldo–Keto Reductase AKR1C1–AKR1C4: Functions, Regulation, and Intervention for Anti-cancer Therapy

**DOI:** 10.3389/fphar.2017.00119

**Published:** 2017-03-14

**Authors:** Chen-Ming Zeng, Lin-Lin Chang, Mei-Dan Ying, Ji Cao, Qiao-Jun He, Hong Zhu, Bo Yang

**Affiliations:** Zhejiang Province Key Laboratory of Anti-cancer Drug Research, Institute of Pharmacology and Toxicology, College of Pharmaceutical Sciences, Zhejiang UniversityHangzhou, China

**Keywords:** aldo–keto reductases, catalytic-dependent, catalytic-independent, inhibitor, therapy

## Abstract

Aldo–keto reductases comprise of AKR1C1–AKR1C4, four enzymes that catalyze NADPH dependent reductions and have been implicated in biosynthesis, intermediary metabolism, and detoxification. Recent studies have provided evidences of strong correlation between the expression levels of these family members and the malignant transformation as well as the resistance to cancer therapy. Mechanistically, most studies focus on the catalytic-dependent function of AKR1C isoforms, like their impeccable roles in prostate cancer, breast cancer, and drug resistance due to the broad substrates specificity. However, accumulating clues showed that catalytic-independent functions also played critical roles in regulating biological events. This review summarizes the catalytic-dependent and -independent roles of AKR1Cs, as well as the small molecule inhibitors targeting these family members.

## Introduction

The AKR1C1–AKR1C4 genes are located on chromosome 10 p15-p14 and comprise of 12 exons. And the average molecular weight of enzymes is estimated to be 34–42 kDa. These enzymes share a high percentage of amino-acid sequence identity that ranges from 84 to 98%. In particular, AKR1C1 and AKR1C2, differ by only seven amino-acid residues (Jez et al., 1997).

The AKR1C isoforms play pivotal roles in NADPH dependent reductions. Therefore, the enzymes are highly related to malignant cancer involve NADPH reductive progress like PCa, breast cancer, and etc. Whereas, discoveries about the catalytic-independent role of the AKR1C isoforms, has also been revealed, including their function as a coactivator, regulation in E3-ligase-ubiquirin system, cell sensitivity, apoptosis, and metastasis.

## AKR1C1–C4-Hydroxysteroid Dehydrogenase

AKR1C isoforms catalyze NADPH dependent reductions at the C3, C5, C17, and C20 positions on the steroid nucleus and side-chain and act as 3-keto-, 17-keto-, and 20- ketosteroid reductases to varying extents in humans (Rižner and Penning, 2014).

AKR1C4 is mainly liver-specific (Deyashiki et al., 1994) and recently it has been proved to be related to manic/hypomanic irritability in males (Johansson et al., 2011, 2012). AKR1C4 efficiently catalyzes the reduction of 5α-pregnane-3,20-dione to yield 3α-hydroxy-5α-pregnan-20-one (allopregnanolone) which is the precursor of and rosterone (Higaki et al., 2003).

The AKR1C3 protein is also known as PGF synthase that catalyzes the conversion of prostaglandins H2 and D2 into PGF2α and 9α,11β-PGF2α respectively (Suzuki-Yamamoto et al., 1999). It has the highest catalytic efficiency of the AKR1C enzymes to interconvert testosterone with Δ^4^-androstene-3,17-dione (Sharma et al., 2006). The enzyme will also reversibly reduce 5α-DHT, estrogen and progesterone to produce 3α-androstanediol, 17β-estradiol and 20α-hydroxprogesterone, respectively (Penning et al., 2001). There are also significant correlations between the expression levels of AKR1C3 and CRPC. And AKR1C3 overexpression is proved to be a promising biomarker for PCa progression (Tian et al., 2014; Hagberg Thulin et al., 2016). Positive AKR1C3 immunoreactivity was also extensively present in both adenocarcinoma and squamous cell carcinoma arising from the lung and the gastroesophageal junction (Miller et al., 2012). Strong correlations between AKR1C3 and tumors were also demonstrated in human colorectal cancer (Hanada et al., 2012; Nakarai et al., 2015), columnar epithelium (Miller et al., 2012), and endometriosis (Sinreih et al., 2015a; Gibson et al., 2016).

While AKR1C4 and AKR1C3 are almost exclusively in the liver and prostate respectively, AKR1C1 and AKR1C2 are most prominent in the mammary glands includes breast cancer, endometrial cancer, colorectal cancer (Hanada et al., 2012; Hofman et al., 2015; Sinreih et al., 2015b; Wang et al., 2016; Wenners et al., 2016). AKR1C2, is also known as bile-acid binding protein and DD2, has lower catalytic efficiencies but preferentially reduces 3-ketosteroids. AKR1C2 preferentially reduces DHT to the weak metabolite 5α-androstane-3α,17β-diol (3α-diol) without conversion of 3α-diol to DHT in the PC-3 cell line (Ji et al., 2003). Progesterone is found to be essential for maintenance of early pregnancy (Agrawal and Hirsch, 2012) and blunting estrogen signaling in endometrial cancer (Ham et al., 1975). And AKR1C1 is the predominant 20-ketosteroid reductase in man and play an important role in reductive inactivation of progesterone into 20α-DHP (Rižner et al., 2006).

## Catalytic-Dependent Biological Role and Cancer

### DHT and Prostate Cancer

Prostate cancer is the most commonly diagnosed solid tumor and the second cause of cancer-related mortality (Tanaka et al., 1993). Androgens drive PCa cell growth via the AR. Accordingly, ADT has been the mainstay in the treatment of advanced PCa patients. However, patients eventually relapse and develop into the lethal form of the disease, termed CRPC (Zong and Goldstein, 2013; Zhang et al., 2016a).

Recent evidence suggests that CRPC may be caused by augmented androgen/AR signaling, generally involving AR overexpression (Yuan and Balk, 2009; Taylor et al., 2010; Shiota et al., 2011). Therefore, newer therapies that target androgen metabolizing AR are being developed and have shown clinical efficacy, indicating the continued importance of the androgen signaling axis in advanced PCa (Higano and Crawford, 2011).

AKR1C3 plays an important role for the biosynthesis of testosterone and estradiol. Elevated levels of AKR1C3 expression in CRPC over PCa have been reported (Tian et al., 2014). The differential distribution of AKR1C isoforms includes AKR1C1 and AKR1C2 has been implicated in the maintenance of a pro-estrogenic or a pro-androgenic state which contributes to development of CRPC as well (Hofland et al., 2010).

High affinity binding of DHT to the AR initiates androgen-dependent gene activation and contributes to PCa development and progression. DHT is synthesized predominantly by 5α-reduction of testosterone (5α-DHT) (Mohler et al., 2011).

In the prostate, 5α-DHT can be reduced to 3α-diol through the action of reductive 3α-HSDs. Between the two major 3α-HSD isozymes, AKR1C2 and AKR1C3, in human prostate, both isozymes catalyze the reversible reduction of 5α-DHT activity toward the weakly androgenic metabolite 3α-diol, which is recognized as a weak androgen with low affinity toward the AR. AKR1C1, which is associated with the HSD3B pathway of DHT metabolism, expressed at higher levels than AKR1C2, catalyzes the irreversible conversion of DHT to 3β-diol (Zhang et al., 2016a). Therefore, the 3α-HSD regulate the occupancy of the AR (Ji et al., 2003; Yepuru et al., 2013).

Recent study has found a first-in-class orally available inhibitor of AKR1C3, ASP9521, which demonstrated anti-tumor activity *in vitro* and *in vivo* preclinical models (Loriot et al., 2014). SN33638, a selective inhibitor of AKR1C3, can prevent the conversion of PGD2 to11β-PGF2α. However, due to the involvement of additional enzymes in testosterone and 17β-estradiol synthesis, its activity at preventing steroid hormone reduction and resultant CRPC and ER-positive breast cancer growth is limited to small subpopulation of CRPC patients with tumors that have upregulated AKR1C3 expression and are dependent on AKR1C3 for producing the testosterone required for their growth (Yin et al., 2014).

### Progesterone and Breast Cancer

Breast cancer is the most frequently diagnosed cancer in women worldwide. The ovarian steroid hormone, progesterone, and its nuclear receptor, the progesterone receptor, are implicated in the progression of breast cancer (Ross et al., 2000). Progesterone binding to its receptor supports an increased progesterone-responsive gene expression and therewith tumor growth and progression (Ji et al., 2004).

AKR1C3 is known to be abundantly expressed in breast cancer tissues, and high levels are often associated with adverse clinical outcome. AKR1C3 is capable to produce intratumorally testosterone and 17β-estradiol by reducing the androgen precursors and estrogen, respectively. The local conversion of less potent hormones to more potent ones will lead to nuclear receptor activation and tumor progression. Therefore, AKR1C3 has recently been identified as a potential therapeutic target in both CRPC and ER-positive breast cancer. AKR1C3 is responsible for the reduction of PGD2 to11β-PGF2α, both of which were reported to demonstrate similar affinities toward their cognate receptor, Prostaglandin receptor (FP receptor). And the action of FP receptor ligands results in carcinoma cell survival in breast cancer (Yoda et al., 2015). AKR1C3 is also associated with doxorubicin resistance in human breast cancer (Zhong et al., 2015).

However, a large proportion (about 30–60%) of breast tumors are PR negative (McGuire et al., 1982; Taucher et al., 2003; Rexhepaj et al., 2008), and about 90% of normal proliferating breast epithelial cells are receptor negative (Robinson et al., 2000). Patients with receptor-negative tumors do not respond to current steroid hormone-based therapies and generally have significantly higher risk of recurrence and mortality compared with patients with tumors that are ER- and/or PR-positive (Wiebe et al., 2013). Overall, this means that for receptor-negative breast cancers, current explanations based on estrogen and progesterone actions and receptors are inadequate, and the related hormone-based therapies are ineffective. Therefore, it is critical to reveal the potential mechanism in regulating breast cancer.

The expression of AKR1C1 and AKR1C2 was found reduced in tumorous breast tissue (Lewis et al., 2004). Then *in vitro* studies had shown that progesterone metabolites can regulate PR-negative breast cell tumor formation and growth as well as tumor regression and maintenance of normalcy. Progesterone is degraded to its metabolite 20α-DHP by AKR1C1 and to 3α-HP by AKR1C2. These metabolites promote suppression of cell proliferation and adhesion. These 20α-DHP and 3α-HP bind to specific plasma membrane receptors, separate from classical HRs, and influence anti-proliferative functions on mitosis, apoptosis, and cytoskeletal and adhesion molecules (Lewis et al., 2004). Evidence has also been presented that progesterone metabolites, 5αP exhibits pro-cancer effects.

### Drug Resistance

Resistance to anticancer drugs and organ specific toxicity are two of the major problems in chemotherapy. Although this phenomenon has been repeatedly observed in the experimental setting, to our knowledge it has not been clinically exploited. An emerging theme is the role of AKRs in cancer chemotherapeutic drug resistance (Barski et al., 2008). And the induction of AKRs was found to be correlated with changes in drug’s properties.

Among the mechanisms of resistance, metabolic inactivation by carbonyl reduction is a major cause of chemotherapy failure that applies to drugs bearing a carbonyl moiety. Oracin is a promising potential cytostatic drug which is presently in phase II clinical trials. Continuously studies found that AKR1C1, AKR1C2, and AKR1C4 mediate the carbonyl reduction of the novel anticancer drug oracin (6-[2-(2-hydroxyethyl)-aminoethyl]-5,11-dioxo-5,6-dihydro-11H-indeno [1,2-c]isoquinoline) to its inactive metabolite DHO (Wsol et al., 2007; Novotna et al., 2008).

AKR1C3 does also catalyze the inactivation of the anticancer drug doxorubicin. Doxorubicin undergoes metabolic detoxification by carbonyl reduction to the corresponding C13 alcohol metabolite, doxorubicinol (Minotti et al., 2004). In comparison to doxorubicin, doxorubicinol exhibited dramatically reduced cytotoxicity, reduced DNA-binding activity, and strong localization to extra nuclear lysosomes (Heibein et al., 2012). Induction of AKR1C1 and AKR1C3 has been shown to efficiently abolish the efficacy of daunorubicin chemotherapy for leukemic U937 cells by metabolizing both DNR and cytotoxic aldehydes derived from ROS-linked lipid peroxidation (Matsunaga et al., 2014). Aldo–keto reductase 1C3 (AKR1C3) is also linked to doxorubicin resistance in human breast cancer which resulted from activation of anti-apoptosis PTEN/Akt pathway via PTEN loss (Zhong et al., 2015). And the reduction of daunorubicin and idarubicin, which is catalyzed by AKR1C3, also contributes to the resistance of cancer cells to anthracycline treatment (Hofman et al., 2014).

The biochemical basis for resistance to cisplatin in a human ovarian cancer cell line has also been reported to be due to overexpression of the AKR1C1 though the underlying mechanism has not been revealed yet (Deng et al., 2002). Knockdown of both AKR1C1 and AKR1C3 in the resistant cells or treatment of the cells with specific inhibitors of the AKRs increased the sensitivity to cisplatin toxicity (Matsunaga et al., 2013).

## Catalytic-Independent Biological Role and Cancer

### Coactivator

Previous studies about AKR1C isoforms mostly revealed their biological function in an catalytic-dependent role. However, their non-catalytic functions have remained elusive until *Yepuru M.* found that AKR1C3 can function as an AR-selective coactivator.

Early studies presented that AKR1C3 catalyzes the adrenal androgens into testosterone, which binds to AR or get converted to DHT, resulting in ligand occupancy of AR. Therefore, AKR1C3 is proposed to play a vital role in the emergence of CRPC by activation of its enzyme activity.

**FIGURE 1:**
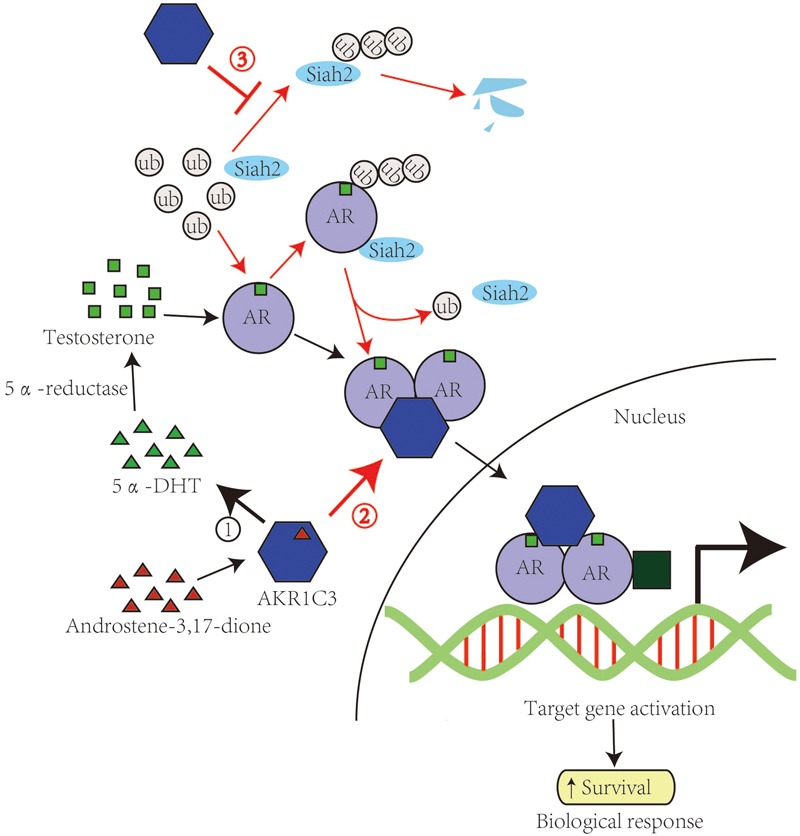
**AKR1C3 promotes PCa via catalytic-dependent and independent roles.** The catalytic and catalytic-independent functions of AKR1C3 in the progression of PCa are shown in black arrows and red arrows respectively. AKR1C3 catalyzes androstene-3,17-dione into 5α-DHT, which is reduced into testosterone by 5α-reductase and binds to androgen recptor. AKR1C3 can also bind to dimerizated and phosphorylated androgen receptors and function as a coactivator of AR. AKR1C3 is able to stabilize Siah2 and thus enhance AR transcriptional activity.

Notably, it was recently reported that AKR1C3 can regulate AR activity in a catalytically independent role. *Yepuru M*. and his co-workers found that as an enzyme converts androstenedione to testosterone, AKR1C3 also acts as a selective coactivator for the AR to promote CRPC growth. AR can interact with AKR1C3 and get recruited to the ARE on the promoter of androgen responsive genes. Thus, recruits related cofactors leading to activation of transcription on reduction of target genes. And while the full-length of proteins is necessary to mediate AKR1C3’s enzymatic functions, amino acids 171–237 were sufficient to mediate the AR activation. These observations identify AKR1C3 a high priority target in PCa progression, considering its dual role as a coactivator and androgen biosynthetic enzyme (**Figure 1**).

### E3-Ligase-Ubiquitin System Regulation

Another example of a catalytically independent role of AKR1C3 on AR activity was found in regulating Siah2 stability. Ubiquitin ligase Siah2 was reported to enhance AR transcriptional activity and PCa cell growth (Qi et al., 2013). Further study found that AKR1C3 shows the ability to bind and stabilize Siah2 by blocking Siah2 self-ubiquitination and degradation (Fan et al., 2015).

Interactions between steroid biosynthetic enzymes and steroid receptors may be exceedingly complex and involved in a variety of hormone-dependent cancers (Yepuru et al., 2013). Future clinical trials with AKR1C3 inhibitors will be needed to show their potential to be the next generation of tissue-specific therapeutics for CRPC. Therefore, identification of mechanisms underlying the non-catalytic function of AKR1C3 may provide new targets for development of novel AKR1C3 inhibitors that complement inhibitors targeting AKR1C3 catalytic activity as potential CRPC therapy (**Figure 1**).

### Cell Sensitivity, Growth, Metastasis, and Apoptosis

The AKRs were also found to be implicated in cell sensitivity, growth, metastasis, and apoptosis in a catalytic independent role, though the underlying mechanisms are still not revealed yet.

Firstly, there was evidence that after short-term and long-term cadmium exposure, the expression of AKR1C1 was elevated which implies the role of ARKs in cell sensitivity (Garrett et al., 2013). Then studies found that AKR1C3 siRNA significantly enhanced cell radio sensitivity (Xie et al., 2013). Consistently with this, overexpression of AKR1C3 enhances resistance of cancer cells to radiation (Xiong et al., 2014; Sun et al., 2016). Participation of AKR1C3 in cancer development is also well proven. Down-regulation of AKR1C3significantly decreases PCa and MCF7 breast cancer cell growth (Downs et al., 2011; Zhang et al., 2016b). Besides, silencing of AKR1C3 increases LCN2 expression and inhibits metastasis in cervical cancer (Wu et al., 2014). AKR1C2 is mostly involved in the process of metastasis. Li et al. (2016) identified two powerful genes in the liver cancer metastasis process, AEG-1 and AKR1C2. And then AEG-1 was proved to promote metastasis through downstream AKR1C2 and NF1 in liver cancer (Li et al., 2014b, 2016). Since AEG-1 and AKR1C2 promote metastasis, inhibiting those two genes would effectively control metastasis.

These findings may provide novel potential clinical targets against metastasis in liver cancer patients. Notably, AKR1C2 is also involved in apoptosis induced by Panax ginseng polysaccharide (Li et al., 2014a).

## Small Molecule Inhibitors

Several types of AKR1C1 inhibitors have been identified, including, benzodiazepines, steroid carboxylates, phytoestrogens, derivatives of pyrimidine, phthalimide, anthranilic acid and cyclopentane, flavones and ruthenium complexes (Usami et al., 2002; Bauman et al., 2005; Brozic et al., 2006b, 2009; Stefane et al., 2009; Liu et al., 2011; Traven et al., 2015). Notably, 3-bromo-5-phenylsalicylic acid, an inhibitor designed based on the structure of AKR1C1 in ternary complex with NADP^+^ and DCL, its phenyl group targets a non-conserved hydrophobic pocket in the active site of the enzyme lined by residues Leu54, Leu308 and Phe311, resulting in a 21-fold improved potency (*K*_i_ = 4 nM) over the structurally similar AKR1C2 (Carbone et al., 2009). Moreover, compound 3-bromo-5-phenylsalicylic acid significantly decreased the metabolism of progesterone in the cells with an IC_50_ value of 460 nM.

Structure between AKR1C1 and AKR1C2 is rather similar, only differs by one active-site residue (Leu54 versus Val54). Therefore, the selectivity of inhibitors targeting AKR1C1 and AKR1C2 is rather low, and newly designed inhibitors that mostly interact with Leu54 in AKR1C1 are needed as to improve the selectivity over AKR1C2. Derivatives of BPSA, 3-chloro-5-phenylsalicylic acid (*K*_i_ = 0.86 nM), is 24-fold more selective for AKR1C1 over AKR1C2. Furthermore, the compound potently inhibited the metabolism of progesterone by AKR1C1 in the cells with an IC_50_ value of 100 nM (El-Kabbani et al., 2010).

AKR1C3 is inhibited by several classes of AKR1C3 inhibitors, including cinnamic acid (Brozic et al., 2006a), non-steroidal anti-inflammatory drugs (NSAIDs) and their derivatives (Gobec et al., 2005; Byrns et al., 2008; Liedtke et al., 2013), steroid hormone analoges (Bydal et al., 2009), flavonoids (Skarydova et al., 2009), cyclopentanes (Stefane et al., 2009), benzoic acids (Adeniji et al., 2011; Jamieson et al., 2012), progestins (Beranic et al., 2011), baccharin analogs (Zang et al., 2015), ruthenium complexes (Kljun et al., 2016), and the most widely used anti-diabetes drugs, sulfonylureas (Zhao et al., 2015). Most inhibitors of AKR1C3 are carboxylic acids, whose transport into cells is likely dominated by carrier-mediated processes. Therefore, development of non-carboxylate inhibitors of AKR1C3 like 1-(4-(piperidin-1-ylsulfonyl)phenyl)pyrrolidin-2-ones (Heinrich et al., 2013) and morpholylureasis essential (Flanagan et al., 2014).

Critical concern in exploiting AKR1C3 inhibitors is the cross inhibition of AKR1C subfamily members, as they have high amino acid sequence identity and structural similarity. This prompts us to find new inhibitors with new molecular skeleton or binding domains (**Table 1**).

**Table 1:** Small molecular inhibitors.

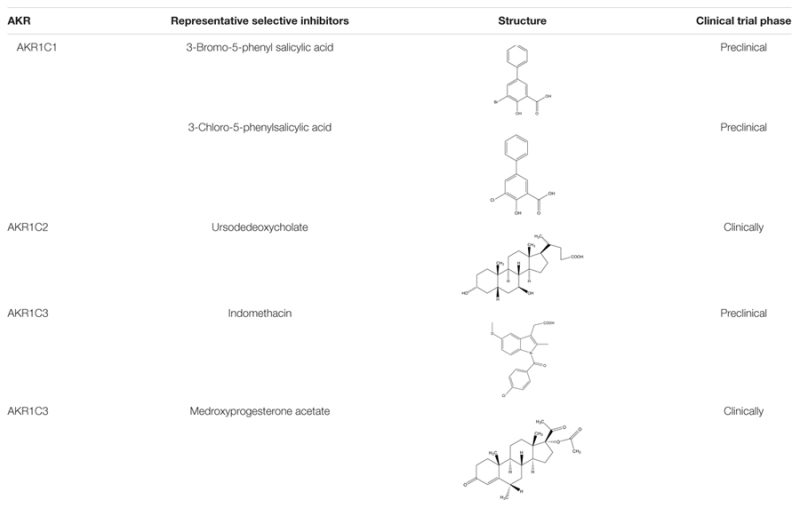

## Perspective

The aldo–keto reductases AKR1C1–AKR1C4 is a series of four proteins with a multitude of functions. Recent advances have been made in terms of the roles played by this family among a variety of diseases, particularly, those functions related to their catalytic activities. However, some clues showed that the catalytic-independent functions of these proteins are totally arousing as well, and aiding in highlight the AKR1C family as promising anti-cancer targets for cancer treatment. Nonetheless, exploration of more potent AKR1C-targeting strategies to interrupt their catalytic activities or the other critical functions is still in urgent need. We rest assured that future developments in this area will absolutely enrich our understanding of the AKR1C isoforms and provide new avenues for using this knowledge to improve cancer therapy (**Table 2**).

**Table 2:** Role of human AKRs in health and disease.

AKR	Associated disease	Selective inhibitors	Clinical trial phase
AKR1C1	Colorectal cancer Breast cancer Endometrial cancer Pre-term birth NCSCL	3-Bromo-5-phenyl salicylic acid	Preclinical
AKR1C2	Androgen insufficiency	Ursodedeoxycholate	Preclinical
AKR1C3	HPRC Breast cancer Acute myeloid leukemia NSCLC	Indomethacin 6-medroxyprogesterone acetate	Clinically
AKR1C4	Paranoia		Preclinical

## Author Contributions

BY and HZ conceived, designed the conception of review article, and made the amendments of the paper. C-MZ conducted the paper. L-LC, M-DY, JC, and Q-JH collected the related research articles.

## Conflict of Interest Statement

The authors declare that the research was conducted in the absence of any commercial or financial relationships that could be construed as a potential conflict of interest.

## Abbreviations

3α-HP: 3α-hydroxyprogesterone
5αP: 5α-dihydroprogesterone
20α-DHP: 20α-dihydroprogesterone
ADT: androgen deprivation therapy
AKR1C isoforms: Aldo–keto reductases
AR: androgen receptor
CRPC: castration-resistant prostate cancer
DHO: dihydrooracin
DHT: dihydrotestosterone
ER: estrogen receptor
HR: hormone receptors
PCa: prostate cancer
PGF: prostaglandin factor
PR: prostaglandin receptor
